# Association between contextual factors and coverage of the Acwy meningococcal vaccine, after three years of its overdue, in the vaccination calendar of adolescents in the state of Minas Gerais, Brazil: global space regressions

**DOI:** 10.1186/s12879-023-08549-6

**Published:** 2023-09-19

**Authors:** Josianne Dias Gusmão, Thales Philipe Rodrigues da Silva, Gustavo Velasquez-Melendez, Larissa Loures Mendes, Milene Cristine Pessoa, Sheila Aparecida Ferreira Lachtim, Mariana Santos Felisbino-Mendes, Luana Carolina Santos, Gilmar José Coelho Rodrigues, Aline Mendes Vimieiro, Ed Wilson Rodrigues Vieira, Fernanda Penido Matozinhos

**Affiliations:** 1 Secretaria Estadual de Saúde de Minas Gerais, Belo Horizonte, Brazil; 2 Post Doctoral Resident. Graduate Nursing Program, School of Nursing, Universidade Federal de Minas Gerais, Belo Horizonte, Minas Gerais, Brazil; 3 Department of Maternal and Child Nursing and Public Health, School of Nursing, Universidade Federal de Minas Gerais, Belo Horizonte, Brazil; 4 Nutrition Department, School of Nursing, Universidade Federal de Minas Gerais, Belo Horizonte, Brazil

**Keywords:** Meningococcal meningitis, Vaccine-preventable diseases, Adolescents, Epidemiology, social environment

## Abstract

The objective of the study was to analyze the spatial distribution of vaccination coverage of bacterial meningitis vaccine: A, C, W and Y (menacwy) and identify the association between socioeconomic and social environment factors with menacwy vaccine coverage among adolescents in the state of Minas Gerais (MG), Brazil. This is an ecological, mixed study, conducted with secondary data from the 853 municipalities of the State of MG, Brazil, from 2020 to 2022, provided by the information system of the National Immunization Program. For spatial statistical analysis, spatial dependence and the presence of spatial clusters formed by municipalities with high and low vaccination coverage of Menacwy were evaluated. In the year 2021, MG presented the largest vaccination coverage (60.58%) since the introduction of the Menacwy vaccine by the PNI. Regarding the analysis of global regressions, it is observed that for the year 2020, as the MG Index of Social Responsibility-Health increased and MG Index of Social Responsibility—Public Security increased, increased the vaccination coverage of the municipalities of the Menacwy vaccine. Finally, compared to 2021, similar association was observed in relation to the proportion of the population served by the Family Health Strategy of the municipalities of the state of MG and per capita spending on education activities: as this indicator increased, with increased coverage of the Vaccine of the Menacwy vaccine of the state municipalities. They reinforce the importance of assessing the quality-of-care management and health surveillance system, professional training, and damage reduction to populations, especially adolescents.

**Supplementary Information:**

The online version contains supplementary material available at 10.1186/s12879-023-08549-6.

## Background

Vaccination is one of the great achievements in public health in history [1] and is a priority, effective and strategic action of primary health care (PHC) [2, 3]. Immunization programs contribute to the improvement of quality and increasing the expectation of world life, due to the control of immunopreventable diseases through vaccines [3]. In the Brazilian scenario, the National Immunization Program (PNI), created in 1973 and coordinated by the Ministry of Health, is recognized worldwide for providing the Brazilian population free access to vaccination, and stands out for its degree of complexity, a since the number of immunobiologicals offered is high and there is a diversification of vaccine schemes [3]. Diseases that until then were controlled returned to affect the Brazilian population [2]. In this context, adolescents stand out, for which the lowest vaccination coverage rates are observed [4–7].

Among the vaccines advocated in the adolescent vaccination schedule, the incorporation in the PNI, in 2020, for adolescents aged 11 to 12, as a booster dose, the vaccine against meningococcus Acwy, a conjugate immunizer that protects against four serogroups of Bacterial meningitis: A, C, W and Y (menacwy) [8, 9].

Meningococcal disease (MD) is a rare but severe disease caused by bacteria *Neisseria Meningitidis* s [10]. It presents itself with fast onset, high lethality rate and substantial irreversible sequelae among survivors and, therefore, the prevention of meningococcal disease remains a public health priority [10, 11]. At least 12 serogroups of *Neisseria Meningitidis* have been identified, among which serogroups A, B, C, W and Y are responsible for almost all the load attributed to meningococcal disease [11].

Given the severity of the disease, the recent introduction of vaccination against meningococcus Acwy in the Brazilian PNI calendar, the growing vaccination hesitation movement in the world and in Brazil and the problem of vaccination coverage reduction involving adolescents, to evaluate the process of introducing the vaccine Menacwy and identify factors that can influence vaccine coverage are essential to trace effective strategies to expand coverage and ensure protection as many individuals as possible.

The acceptability of vaccines is a complex process that can be affected by several factors, such as contextuals, specific vaccine issues and directly related to their characteristics or vaccination process and individual and group aspects [12].

Achieving vaccine coverage goals, among the teenagers is a challenge [13, 14], as several reasons can justify low vaccination coverage between this population group. According to the theoretical model described by Behary et al. [14], this phenomenon can be justified for reasons grouped on three major organizational levels, namely: reasons related to the user/patient (adolescent), reasons related to professionals who provide care to adolescents and reasons related to the organization of health service and social structures. Study conducted in the state of Minas Gerais, state of the southeastern region of Brazil, showed that factors of the social environment, such as the violence rates in the municipalities, interfere with the vaccination coverage of adolescents [7].

This study aimed to identify the association between socioeconomic and social environment factors with Menacwy vaccine coverage rates among adolescents in the state of Minas Gerais (MG), Brazil.

## Methods

This is an ecological study, conducted with secondary data from the State of Minas Gerais, Brazil, from 2020 (year of introduction of the vaccine) to 2022, made available by the Information System of the National Immunization Program (SI-PNI), available at < http://sipni.datasus.gov.br/ > , about the menacwy vaccine.

The State of Minas Gerais consists of 853 municipalities, a state with the largest number of municipalities in Brazil, distributed in a territory of 586,528 km^2^, with a population of 21,168,791 inhabitants in 2019. The state was divided into 14 expanded regions Health, macro-regions considered as a territorial basis for health care planning, due to its demographic, socioeconomic, geographical, sanitary, epidemiological characteristics, supply of healthcare services and relations between municipalities. They are: South; South Center; Center; Jequitinhonha; West; East; Southeast; North; Northwest; Southern east; North East; Southern triangle; Northern triangle and; Steel Valley.

The State of Minas Gerais is also divided into 19 Regional Health Superintendencies (SRS) and 9 Regional Health Management (GRS). Municipalities are delimited from cultural, economic, and social identities and shared transport communication and infrastructure networks, with the purpose of integrating the organization and planning of health actions and services.

## Data collection

### Outcome

The menacwy vaccine, the variable dependent on this study, was introduced at the PNI in 2020 for adolescents between the 11–12 years age group and, in the year 2022 [8], due to the scenario experienced by the Covid-19 pandemic, the age group was expanded for 11 to 14 -year -old teenagers [15]. For the public in question, the menacwy vaccine is administered in a single dose scheme [8].

For the calculation of vaccination coverage (VC), the resident population of the target age group was considered: 2020 and 2021, adolescents from 11 to 12 years and, for the year 2022, due to the expansion of the age group of the adolescents and unavailability of the data for the year, the population of 10 to 13 years in the year 2021, that is, that in 2022 would be in the age group of 11 to 14 years. The target population was extracted from the study of population estimates by municipality by age and gender of the years 2000–2021 of Brazil, available at < http://tabnet.datasus.gov.br/cgi/deftohtm.exe?ibge/cnv/popsvsbr.def > .

For the numerator, the applied doses of the menacwy vaccine were used in adolescents of the target population, according to the national vaccination calendar of the Ministry of Health. The cohort methodology of vaccinated adolescents was applied, that is, for the year 2020 only the doses of the menacwy vaccine applied in adolescents aged 11 to 12 years. Regarding the year 2021, the applied doses of the menacwy vaccine applied to adolescents who were 11 years old in 2020 (teenagers were 12 years old in 2021) adding the doses applied in the year 2021 in adolescents who composed the age group. To generate the coverage of the year 2022 the doses applied to adolescents of 11 and 12 years in 2020 (the teenagers were 13 and 14 years old in 2022, respectively), with doses applied in 2021 (the teenagers passed to be 12 and 13 years old in 2022), in addition to the doses of the Menacwy vaccine effectively administered in the year 2022 for the target population, i.e. adolescents between 11 and 14 years.

In the present study, because it is a single dose vaccine, three eligible cohorts were defined for vaccination in 2020 to 2022 (Table 1).

**Table 1:** Age-Period-Cohort that specifies the clipping of the population used for the calculation of vaccination coverage against meningococcus Acwy, Minas Gerais, Brazil

2020	2021	2022*
9 years	10 years	11 years – C3
10 years	11 years – C2, C3	12 years – C3
11 years – C1, C2, C3	12 years – C2, C3	13 years – C3
12 years – C1, C3	13 years- C3	14 years—C3

C1, C2, C3 = teenage cohort per year

### Exposures

As independent variables of this study, the sociodemographic variables of the municipalities detailed in the Supplementary material 1 (Table S1) and available on the João Pinheiro Foundation website were adopted < http://imrs.fjp.mg.gov.br/Consultas > , called Minas Gerais Index of Social Responsibility (IMRS). IMRS, was created by State Law No. 15,011 of 2004, which defined that it should be calculated by the João Pinheiro Foundation (FJP) for all municipalities of the state [16]. For this study, the hypothesis was that socio-economic factors may be associated with compliance with PNI goals for adolescents, namely: Minas Gerais Index of Social Responsibility (IMRS), Minas Gerais Index of Social Responsibility—Social Assistance (ImRSSSIST), Minas Gerais Index of Social Responsibility—Education (IrSeduca), Minas Gerais Index of Social Responsibility—Health (Imbsaude), Minas Gerais Index of Social Responsibility—Public Safety (IMRSEGP), Minas Gerais Index of Social Responsibility—Vulnerability (Impersvulnera), Minas Gerais Responsibility Index Social—Sport, Tourism and Leisure (IMRESPTL), High School Net Schooling Rate (E_TAXA15), Gross Standard Mortality Rate (S_TXBrutamortPad), Total Population Homicide Mortality Rate (S_TXMOHOMI), proportion of the population served by the strategy of Family Health (S_COBPSF), proportion of hospitalizations for conditions sensitive to Primary Health Care (s_ICSAB_MS), percentage of the poor or extremely poor population in the CadÚnico (*Cadastro Único,* it is an instrument that identifies and characterizes low-income families*.* CadÚnico constitutes an important support tool for the formulation and implementation of public policies capable of promoting the improvement of the lives of these families, as it gathers a set of data that allows managers to know the risks and vulnerabilities to which the poor and extremely poor population is exposed.) in relation to the total population of the municipality (B_PopPobextrpob), percentage of people 15 years or older who do not know how to read and write and the population in this age group in CadÚnico (B_Penlecad), percentage of poor or extremely poor children or adolescents in the CadÚnico (B_CRIANADOLEXTRPOP), percentage of self -declared brown or black people in the CadÚnico (B_PoppreTapardacads), Urbanization Rate (D_POPURB), per capita spending on education activities (G_education), Violent Crime Rate (P_CV) and Violent Crime Fee Against Person (P_CVPE) (Supplementary Material—Table 1). For the year 2022, indicators are not yet available for consultation.

## Data analysis and processing

### Spatial analysis

Choropleth maps were constructed from the shapes of the 853 municipalities of the state of Minas Gerais, represented by color scales and coverage of the Mencacwy vaccine for the three years of analysis. VC was categorized according to the goals set by the PNI greater than or equal to 80% for immunobiologicals administered in adolescents, being categorized at: very low (0% to < 50%), low (≥ 50% and less than the goal) and adequate (≥ the goal). QGIS software (version 2.18) was used to create the maps.

For spatial statistical analysis, spatial dependence and the presence of spatial clusters formed by municipalities with high and low vaccination coverage of Menacwy were evaluated. The Space Association was analyzed using the Global Moran Index (GMI), which estimates spatial autocorrelation. It was considered direct spatial correlation when GMI > 1, absence of correlation when GMI = 0 and reverse correlation when GMI < 1. For the interpretation of the force of spatial correlation, GMI was classified as weak (< 0.3), moderate (0.3–0.7) or strong (> 0.7) [17].

The presence of spatial conglomerates (clusters) formed by the municipalities of the state of Minas Gerais was evaluated from the analysis of the indicators of spatial association (LISA). Boxmap -type thematic maps were elaborated from the cartographic base containing the limits of the municipalities of Minas Gerais. The Boxmap (Lisa Cluster Map) represents the spatial distribution of the vaccine coverage of the menacwy vaccine in the units of analysis, being the clusters classified in: low-low space clusters (dark blue color), formed by municipalities with low vaccination coverage and surrounded by municipalities with Low vaccination coverage; High-high (dark red color), formed by municipalities with high vaccination coverage and surrounded by municipalities that also had similar behavior; high-low (municipalities with high vaccination coverage surrounded by those with low coverage) and low–high (municipalities with low coverage surrounded by municipalities with high coverage) [17].

In this study, the Moran’s significance level of 95% was considered after 9,999 permutations [17], i.e. the areas with statistically significant spatial correlation were those whose value of P was less than or equal to 0.05 after 9,999 permutations random for both indices.

### Global spatial regression analysis

Initially, multivariate linear regression was applied [18]. For the modeling process, the Backward method was adopted, based on theoretical criteria. Multicollinearity condition number was also observed to identify collinearity of the explanatory variables inserted in the model, as this test evidences collinearity of explanatory variables when its value is greater than 30. The OLS model provides estimates of spatial dependence diagnoses through Langrange Multiplier Tests, which shows the need to consider models that incorporate spatial effects. Subsequently, the models with global spatial effects were performed, which considers spatial effects, namely: spatial lag and spatial error. Through the Langrange Multiplier Tests, the model that presented the highest value of the likes of the likelihood and lower values of the Akaike Information Criteria (AIC) and the Bayesian Schwarz criterion was considered the model of the likes [18, 19].

The spatial lag model does not attribute to the variable response to ignored spatial autocorrelation. In this model, spatial autocorrelation is incorporated as a component of the model itself. The spatial error model, on the other hand, considers spatial effects as noise, i.e. factor to be removed, since the effects of spatial self -coloring are associated with the term of error. Finally, through the Global Moran Index, it was evaluated whether the spatial self -cores of waste was eliminated [18]. Geoda software (version 1.20.0.8) was used for the analysis of this study.

### Ethical approval

Due to the nature of this study of using freely accessible data, available by Information Technology Department of the Brazilian National Health System (Datasus), it was not necessary to submit the present study to the Research Ethics Committee, in accordance with Resolution 466/2012 of the National Brazilian Health Council.

## Results

Between 2020 and 2022, 1,146,071 doses of the menacwy vaccine were applied among the adolescents of the state of Minas Gerais. In the year 2021, Minas Gerais presented the largest vaccination coverage (60.58%) since the introduction of the Menacwy vaccine by the PNI (Table 2).

**Table 2:** Vaccine coverage of Acwy meningococcal vaccine in the state of Minas Gerais among adolescents, per year, 2020 to 2022

Years	Doses applied	Target population	Vaccination coverage (%)
2020	238,086	530,337	**44.89**
2021	316,864	523,033	**60.58**
2022	591,121	1,051,694	**56.21**
**Total**	**1,146,071**		

**Fig. 1:**
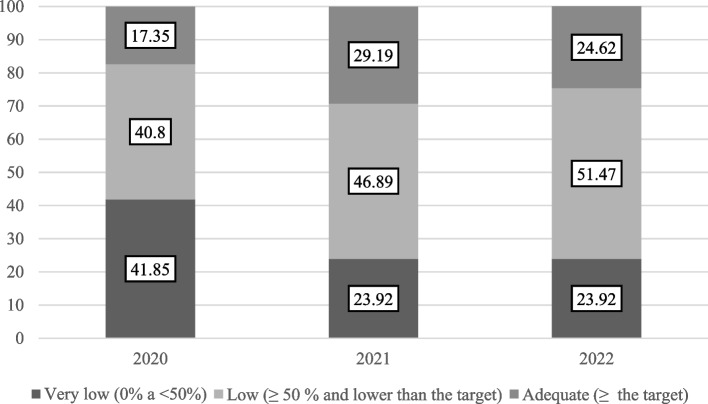
Percentage of municipalities according to classification of vaccination coverage of the Meningococcal ACWY vaccine among adolescents, Minas Gerais, 2020 to 2022 Note: 853 municipalities; target ≥ 80%

The year 2021 presented itself as the year in which the majority (29.19%) of the municipalities reached the target of vaccination coverage and, on the other hand, 2020 presented itself as the year in which most municipalities were classified as very low (41.85%) and low vaccination coverage in the year 2022 (51.47%) (Fig. 1 and supplementary material 2).

There was a spatial autocorrelation of the variable vaccination coverage of the MenACWY vaccine among adolescents in Minas Gerais, by year (2020, 2021 and 2022), and between municipalities using the Moran Global index (year 2020, I = 0.149 and *p*-value < 0.001; year 2021, I = 0.118 and *p*-value < 0.001 and; year 2022, I = 0.128 and *p*-value < 0.001). Through the investigation of the presence of spatial conglomerates (clusters) formed by the municipalities of the state of Minas Gerais, based on the LISA analysis, in 2020, 61 municipalities were identified that made up the cluster of low-low clusters, in 2021 there were 51 municipalities and, in 2022, 58 municipalities made up the low-low cluster. This cluster consisted of municipalities with low vaccination coverage surrounded by other municipalities with low vaccination coverage of the MenACWY vaccine and, in all years, they were located in the region of Triângulo Mineiro, North and South of Minas Gerais (Supplementary Material 3).

The data presented in Table 3 show the vaccination coverage for the MenACWY vaccine by the health macro-regions of Minas Gerais and the proportion of municipalities that make up each one of them and that reached the vaccination coverage targets of 80%. It is observed that in the years 2020, 2021 and 2022, the Jequitinhonha macro-region had the highest vaccination coverage in the state (56.79%, 64.69% and 63.07, respectively). However, when analyzing the percentage of municipalities that made up the macro-regions that reached the target, it was observed that the highest proportion was observed in the East macro-region, in the year 2021, with 28.30%; in the Steel Valley macro-region, with 40.00% and, in the Center-South, in the year 2022, with 37.25% (Table 3).

**Table 3:** Meningococcal ACWY vaccination coverage and percentage of municipalities that achieved the recommended vaccination coverage target of 80% for adolescents in the state of Minas Gerais, according to health macro-regions of Minas Gerais, 2020 to 2022

	**YEARS**					
**Health Macro-region**	**2020**		**2021**		**2022**	
	**Vaccination coverage**	**Municipalities that achieved the goal n (%)**	**Vaccination coverage**	**Municipalities that achieved the goal** **n (%)**	**Vaccination coverage**	**Municipalities that achieved the goal** **n (%)**
Center	43.73	23(22.77)	63.87	37(36.63)	55.83	31(30.69)
South Center	50.12	14(27.45)	61.75	20(39.22)	58.91	19(37.25)
Jequitinhonha	56.79	8(25.81)	64.69	8(25.81)	63.07	10(32.26)
East	51.48	11(21.57)	57.98	13(25.49)	57.49	9(17.65)
South East	42.03	15(28.30)	49.62	16(30.19)	52.31	16(30.19)
North East	52.56	12(21.05)	62.01	19(33.33)	59.57	15(26.32)
North West	41.91	5(15.15)	59.06	7(21.21)	55.77	8(24.24)
North	45.22	10(11.63)	58.72	19(22.09)	55.27	14(16.28)
West	50.53	5(9.43)	65.08	13(24.53)	60.09	9(16.98)
South East	38.03	12(12.77)	50.11	25(26.20)	47.55	23(24.47)
South	47.23	19(12.34)	62.79	50(32.47)	58.90	39(25.32)
Northern Triangle	40.56	1(3.70)	61.70	3(11.11)	59.62	2(7.41)
Southern Triangle	33.67	1(3.70)	49.46	5(18.52)	48.26	3(11.11)
Steel Valley	48.28	12(17.35)	61.68	14(40.00)	58.05	12(34.29)

Table 4 presents the summary of the indices that allow evaluating the quality of the models. When this spatial autocorrelation was introduced into the models, through Spatial Lag and Spatial Error, there was an improvement in the results. However, among the spatial regression methods considered, the results provided by the Spatial Error indicated that this was the model that provided the best adjustment of the studied variables with the highest Log Likelihood value and lowest values of the Akaike Information Criterion (AIC) and of the Bayesian Schwarz criterion (BSC) for both study years (Table 4).

**Table 4:** Ordinary Least Squares Estimation, Spatial Lag and Spatial Error models for vaccination coverage of the Meningococcal ACWY vaccine among adolescents in the state of Minas Gerais, 2020 and 2021

	YEARS												
	**2020**						**2021**						
MODELOS	**OLS**		**Error Lag**		**Spatial Lag**		**OLS**		**Spatial Lag**		**Error Lag**		
	Coefficient	*p*-value	Coefficient	*p*-value	Coefficient	*p*-value	Coefficient	*p*-value	Coefficient	*p*-value		Coefficient	*p*-value
Variables													
**IMRSAUDE**	26.88	**0.0152**	31.93	**0.004**	26.96	**0.0128**	-	-				-	-
**S_COBPSF**	-	**-**	-	**-**			0.24	** < 0.001**	0.23	** < 0.001**		0.23	** < 0.001**
**IMRSSEGP**	44.97	** < 0.001**	52.10	** < 0.001**	45.96	** < 0.001**	-	-				-	-
**G_EDUCACAO**	-	-	-	-			0.009	** < 0.001**	0.009	** < 0.001**		0.009	** < 0.001**
**Langrange multiplier tests**													
Lagrange multiplier (lag)	** < 0.001**						** < 0.001**						
Lagrange multiplier (error)	** < 0.001**						** < 0.001**						
**Robust LM (lag)**	** < 0.001**						0.801						
Robust LM (error)	** < 0.001**						0.331						
Multicollinearity condition number	22.79						15.73						
Log likelihood	-4005.18		-3990.13		-3992.91		-3949.8		-3935.37		-3935.47		
Akaike info criterion	8016.36		7986.27		7993.82		7905.6		7878.75		7876.94		
Schwarz criterion	8030.61		8000.51		8012.82		7919.85		7897.74		7891.19		
R^2^	0.036300		0.082532		0.074704		0.036387		0.080703		0.080497		
Moran (i) – residue	0.0886		-0.014		-0.002		0.0759		-0.004		-0.005		

For the year 2020, it was observed that as the Minas Gerais Social Responsibility Index—Health increased, the vaccination coverage of the municipalities of the MenACWY vaccine increased. As the Minas Gerais Social Responsibility Index—Public Safety increased, the vaccination coverage of the municipalities in the state of Minas Gerais with the MenACWY vaccine also increased (Table 4).

In relation to the year 2021, a similar association was observed in relation to the proportion of the population served by the Family Health Strategy in the municipalities of the state of Minas Gerais, with increased vaccine coverage of the MenACWY vaccine in the state municipalities. Another association found for the year 2021 was in relation to per capita expenditure on education activities: as this indicator increased, so did vaccination coverage for the MenACWY vaccine among adolescents in the state of Minas Gerais (Table 4).

## Discussion

This study showed that since the introduction of the MenACWY vaccine in Brazil, the state of Minas Gerais has not reached the recommended coverage target for adolescents. The year 2021 stands out as the year with the highest number of municipalities that reached the target of 80%.

In 2021, the World Health Organization released the document “Defeating meningitis by 2030”, which aimed to reduce cases of vaccine-preventable bacterial meningitis by 50% and deaths due to MD by 70% [20]. A study carried out in the United Kingdom [21] showed that after three years of the introduction of the MenACWY vaccination program, it was observed: low sustained carrier of serogroup C meningococci; 73% reduction of serogroup W meningococci and a 69% decrease of serogroup Y meningococci, in addition to producing herd protection, reducing DM in all age groups [21]. However, for the year 2018, coverage of the MenACWY vaccine in the cohorts aged 15 to 19 years was 80.95% [21], greater coverage than that found in this study.

The need to reach the recommended goal for reducing DM serogroups is reinforced, but numerous factors influence the vaccination coverage of adolescents. Adolescents rarely attend health services and rarely engage in health promotion activities [22], both due to the characteristics of their biopsychosocial development and the lay understanding that this age group is in “good health” and would not benefit from routine appointments [23]. In addition, many adolescents (and their families) are not aware of the need for vaccination in their age group [5], and the (lack of) health education at school, where adolescents spend most of their time, also contributes to the lack of this knowledge or greater care for your health. Another key point for vaccinating adolescents is professionals, especially FHS professionals. Few professionals in Brazil are prepared to deal with the idiosyncrasies of adolescence [23]. In addition, it is common for professionals not to offer or recommend vaccines to adolescents when they attend appointments at health units [5]. They also use the myth of the need for parents or guardians to provide care/interventions, not guaranteeing the rights of adolescents. Finally, it is known that there are intrinsic reasons to the form of organization of the health system and social constructions, which can impede the path of adolescents to primary care centers [23]. This factor should not be overlooked, especially in a country like Brazil, where 61% of children and adolescents live in conditions of socioeconomic vulnerability. Nor can the role of the COVID-19 pandemic be suppressed in reducing vaccination coverage, both in children, but also among adolescents [24–27].

A study carried out with another immunobiological, exclusively for the adolescent population in Minas Gerais, showed that in 2020 the vaccination coverage rate for this immunobiological was 52.28% for the first dose and 25.69% for the second dose [7]. Another finding of this study is the existence of spatial autocorrelation of the variable vaccination coverage of the MenACWY vaccine among adolescents in Minas Gerais and, in all years, the low-low clusters were located in the region of Triângulo Mineiro, North and South of Minas Gerais. In the Ituiutaba and Pirapora GRS, none of the municipalities in the three years of analysis reached the recommended vaccination coverage target for adolescents. Regarding the contextual factors associated with vaccine coverage of the MenACWY vaccine, it was observed that the variables Minas Gerais Index of Social Responsibility—Health, proportion of the population served by the Family Health Strategy, Minas Gerais Index of Social Responsibility—Public Safety and per capita expenditure capita with education activities influenced the coverage rates for adolescents in the state.

The Minas Gerais Index of Social Responsibility – Health [28] is composed of eight indicators of primary care and hospital medical care, which aim to highlight the health dimension of municipalities in the state of Minas Gerais. This indicator comprises: Mortality Rate due to Chronic Noncommunicable Diseases (CNCD) in the population aged 30 to 69 years (per 100,000 inhabitants), proportion of Hospitalizations due to Conditions Sensitive to Primary Care (ICSAB), Estimate of the Proportion of the population served by the Family Health Strategy (FHS) and Proportion of Hospital Admissions of Medium Complexity of Brazilian Unified Health System (SUS) patients referred to another Health Microregion [28]. Among the indicators that make up the dimension of primary health care, the proportion of ICSAB is commonly used as an indirect measure of quality [28, 29], since high levels of this type of hospitalization suggest problems in the effectiveness of Primary Health Care [28]. The opening hours of health units and the availability of vaccines, for example, are factors that reduce the number of hospitalizations for conditions sensitive to primary care [29, 30]. However, the fact that the vaccine is available does not mean that it will influence the decision of individuals to get vaccinated.

Another important indicator, and also associated in 2021 with the increase in vaccination coverage of the MenACWY vaccine, is the proportion of the population served by the Family Health Strategy of the municipalities. The FHS was officially implemented in Brazil in 1994 and is a milestone in the history of health policies in Brazil, as it reorganized the health care model [31]. In Minas Gerais, the proportion of the population assisted in FHS units was 72.3% in 2013 and, in 2019, it was 73.0% [32]. Through the Secretariat of Primary Health Care of the Ministry of Health (Saps/MS), in May 2019 and with updates with the publication of Ordinance No. 397/GM/MS, of March 16, 2020, the Brazilian Ministry of Health, launched the Health on Time (Saúde na Hora) Program, which provides for the extension of the opening hours of the BHU in the municipalities that adhere to the program, making it easier for the population to access the services offered in primary care, including immunization actions [3].

Another finding of this study was the association of higher vaccination coverage for the MenACWY vaccine in the Minas Gerais municipalities with the highest value in the Minas Gerais Index of Social Responsibility—Public Safety [33]. This indicator is a composite of: Rate of intentional homicides, Rate of violent crimes against property and Inhabitants per military police officer [33]. Crime can limit access to health services for the population and, consequently, influence the quality of health care [34, 35]. In this context, this study shows that important territorial characteristics, materialized through public security policies, economic infrastructure, health, education, and demographic structure, are decisive for the development of public health strategies, especially for vaccination [36, 37].

Finally, there was an association in municipalities that had higher per capita spending on education activities with increases in coverage rates for adolescents in the state, showing the need to think about the intersectoral component of vaccination [38]. The school is an important space to promote constant discussion and education about vaccine events and reduce vaccine hesitancy [39]. Among the strategies that have been tested and proven to be effective for increasing vaccination coverage among adolescents, the most important is increasing access by adolescents to health services, with the organic integration of these services into the means of interaction and sociability of adolescents [40, 41].

It is also noteworthy that the highest vaccination coverage was found in municipalities with small populations, i.e., with populations of less than 20,000 inhabitants. The broad relationship between primary care and small municipalities may be a contributing factor to the efficiency of actions in these cities. According to the National Plan for Basic Care (PNAB) [42], for every 18,000 inhabitants there is a need for a single basic health unit, which brings the population much closer to the professionals. Moreover, community health agents are primary care actors, working through health education, with individual and collective activities, and serving as a link between the professional team and the community [43]. Thus, the smaller the size of the municipalities, the easier it is to communicate, since the distances and the number of people to be approached and informed are smaller. Small-sized municipalities have characteristic aspects that are very favored under SUS operating logic, such as regionalization [44]. Regionalization constitutes a strategy to correct inequalities in access and fragmentation of health services, through the functional organization of the SUS, defining the responsibilities of the federated entities, and the reference flows, to guarantee access for the population residing in the area covered by each regional space. In addition to aspects related to access, efficiency and effectiveness, regionalization strengthens the decentralization process, promoting more cooperative and solidary relationships between SUS managers and qualifying the management capacity of municipal health systems [44]. Therefore, it is possible to believe that the physical proximity imposed by the conditions of these cities was the essential element for the improvement of data in one year [43].

Finally, this study has some limitations, resulting from the use of secondary data and possible “inconsistency” in relation to the quantity and quality of its information. However, it has the potential to support research on issues of importance to the public health of adolescents. It should be noted that a potentiality of this study is the use of the vaccinated cohort methodology, which avoids underestimating vaccine coverage among adolescents.

This article is original and has an innovative character, as it advances from the perspective of analyzing data that has not yet been fully explored on the health of adolescents. The production of works in this area is scarce, mainly those focused on the socioecological determinants associated with the vaccination situation in this life cycle. The use of secondary data enables the identification of associations of social environment factors, which makes the results highly applicable in the improvement not only of health policies, but also reinforces the intersectionality of the conditions that act in the social determination of the health-disease-care process.

## Conclusion

The results of this research show potential factors, such as the social environment, that can influence the ACWY vaccination in adolescents. They reinforce the importance of assessing the quality-of-care management and the health surveillance system, professional training, and the reduction of harm to populations, especially adolescents, as this is a very important care action for primary prevention: vaccination.

These results may provide justification for the development of strategies to prevent injuries and promote health, in addition to interventions, with favorable evidence of the association between school and vaccination, in order to increase vaccination coverage, even in the face of the challenge experienced by the COVID-19 pandemic.

### Supplementary Information

**Additional file 1.**

**Additional file 2.**

**Additional file 3.**

## Data Availability

The datasets used and/or analysed during the present study are available from the corresponding author upon request. Available from: http://tabnet.datasus.gov.br/cgi/dhdat.exe?bd_pni/cpnibr.def.
