# Supplementary material for: Association between contextual factors and coverage of the Acwy meningococcal vaccine, after three years of its overdue, in the vaccination calendar of adolescents in the state of Minas Gerais, Brazil: global space regressions
Source: BMC Infect Dis. 2023 Sep 19;23:615. doi: 10.1186/s12879-023-08549-6 (PMC10507822; doi:10.1186/s12879-023-08549-6)
Supplement: Supplementary file 1 — Additional file 1. [file 12879_2023_8549_MOESM1_ESM.docx]

| **Supplementary material 1**  **Table S1 -** Descriptions of the socio-demographic environment variables - Minas Gerais Index of Social Responsibility (IMRS) | | | | |
| --- | --- | --- | --- | --- |
| **Indicator name** | **Definition** | **Source of data used** | **Available in the year** | |
|  |  |  | **2020** | **2021** |
| Minas Gerais Social Responsibility Index | Weighted average of the subindices referring to ten dimensions: (1) Education; (2) Health; (3) Income and employment; (4) Public Safety; (5) Environment; (6) Sanitation and housing; (7) Culture; (8) Sports, Tourism and Leisure; (9) Social Assistance and (10) Municipal Finance. | João Pinheiro Foundation | **X** |  |
| Minas Gerais Index of Social Responsibility - Social Assistance | IMRS sub-index that seeks to measure social responsibility through situation, effort, and management indicators in the Social Assistance dimension. | João Pinheiro Foundation | **X** |  |
| Minas Gerais Index of Social Responsibility - Education | IMRS sub-index that seeks to measure social responsibility through situation, effort and management indicators in the Education dimension. | João Pinheiro Foundation | **X** |  |
| Minas Gerais Index of Social Responsibility - Health | IMRS sub-index that seeks to measure social responsibility through situation and effort indicators in the Health dimension. | João Pinheiro Foundation | **X** |  |
| Minas Gerais Index of Social Responsibility - Public Safety | IMRS sub-index that seeks to measure social responsibility through situation, effort, and management indicators in the Public Security dimension. | João Pinheiro Foundation | **X** |  |
| Minas Gerais Social Responsibility Index - Vulnerability | IMRS sub-index that seeks to measure social responsibility through indicators that express social vulnerability. It presents indicators that describe the situation of vulnerability to which people or population segments are subject and indicators that represent the effort of the municipality in the area of social assistance. | João Pinheiro Foundation | **X** |  |
| Minas Gerais Social Responsibility Index - Sports, Tourism and Leisure | IMRS sub-index that seeks to measure social responsibility through situation, effort and management indicators in the Sport, Tourism and Leisure dimension. | João Pinheiro Foundation | **X** |  |
| Secondary school net enrollment rate | Ratio between the number of people aged 15 to 17 enrolled in secondary education and the total population in this age group, multiplied by 100. Indicator of access of the population aged 15 to 17 to the educational system of reference for their age group, without take into account age-grade adequacy. | Enrollments: Ministry of Education/INEP; B) Population: IBGE and FJP. | **X** | **X** |
| Crude standardized mortality rate | Ratio between the sum of expected deaths by age group and the total population (estimated by the FJP) of the municipality, multiplied by 1000. For each age group, the expected deaths are obtained by multiplying the specific mortality rate (number of deaths that occurred in this age group divided by the population of the municipality in this age group) by the standardized population of the municipality in this age group (obtained from multiplying the total population of the municipality by the participation of this age group in the population taken as standard), having adopted as a standard the population of the state of Minas Gerais, 2010 (as of 2011). | Deaths: Health Information Tabulator (Tabnet). Health Surveillance and Protection Portal. Minas Gerais State Health Secretariat (SES-MG). Population: In the years 2000 and 2010, IBGE/Demographic Census. In intercensal years, it was estimated by the João Pinheiro Foundation. | **X** | **X** |
| Homicide mortality rate of the total population | Ratio between the number of deaths by homicide (ICD 10: X85-Y09) of residents and the total number of residents, multiplied by 100,000. | Deaths: Health Information Tabulator (Tabnet). Health Surveillance and Protection Portal. Minas Gerais State Health Secretariat (SES-MG). Population: Estimated by the João Pinheiro Foundation. | **X** | **X** |
| Proportion of population served by the Family Health Strategy (S_COBPSF) | Ratio between the service capacity and the total population of the municipality. Service capacity corresponds to the product of the average number of teams in the year (sum of the number of family health teams in each month of the year, divided by 12) and the estimated average service per team, according to SES-MG (3450 people assisted). | PSF teams: State Department of Health of Minas Gerais (SES-MG). Population: Estimated by the João Pinheiro Foundation. | **X** | **X** |
| Proportion of hospitalizations for conditions sensitive to primary care (S_ICSAB_MS) | Ratio between the number of hospitalizations due to conditions sensitive to primary care (considering the MS/SAS Ordinance No. 221, of April 17, 2008, only hospitalizations whose causes are associated with promotion, prevention and even cure and rehabilitation actions, at the primary level of care and that manage, in a short and medium time, to reduce the number of clinical admissions) and the total number of clinical admissions, multiplied by 100. | Department of Primary Care (DAB) - Ministry of Health. | **X** | **X** |
| Percentage of the poor or extremely poor population in the Single Registry in relation to the total population of the municipality (B_POPPOBEXTRPOB) | The indicator refers to the ratio between the poor or extremely poor population registered in the Cadastro Único and the total population of the municipality, multiplied by 100. In the calculation methodology, the following Ministry of Citizenship references were considered for defining the poor or extremely poor population: Poor people - those with per capita income, years 2014 and 2015: from R$ 77.00 to R$ 154.00; years 2016 and 2017: per capita income from R$85.01 reais to R$170.00; years 2018 and 2019: per capita income from BRL 89.01 to BRL 178.00. Extremely poor people - those with per capita income, years 2014 and 2015: less than or equal to R$ 77.00 to R$ 154.00; years 2016 and 2017: per capita income less than or equal to R$ 85.00; years 2018 and 2019: per capita income less than or equal to R$ 89.00/month. Monetary values were updated based on the IPCA, considering the deflator calculated by the ratio of the December index of the current year in relation to the average of the indexes for each year, according to the update date. For the total population of the municipality, population estimates by João Pinheiro Foundation for the period 2014 to 2019 were considered, which are based on IBGE estimates. | Ministry of Citizenship/Secretariat for Evaluation and Information Management - SAGI | **X** | **X** |
| Percentage of people aged 15 or over who cannot read and write and the population in this age group in the Single Registry (B_PENLECAD) | The indicator refers to the ratio between people aged 15 or over who cannot read and write and the population in this age group registered in the municipality's Single Registry, multiplied by 100. | Ministry of Citizenship/Secretariat for Evaluation and Information Management - SAGI. | **X** | **X** |
| Percentage of poor or extremely poor children or adolescents in the Single Registry (B_CRIANADOLEPOBEXTRPOP) | The indicator refers to the ratio between the number of children or adolescents belonging to poor or extremely poor families registered in the Single Registration and the total number of children or adolescents in families registered in the Single Registration of the municipality, multiplied by 100. In the calculation methodology, it considered the following references from the Ministry of Citizenship are used to define the poor or extremely poor population: Poor people - those with per capita income, years 2014 and 2015: from R$ 77.00 to R$ 154.00; years 2016 and 2017: per capita income from R$85.01 reais to R$170.00; years 2018 and 2019: per capita income from BRL 89.01 to BRL 178.00. Extremely poor people - those with per capita income, years 2014 and 2015: less than or equal to R$ 77.00 to R$ 154.00; years 2016 and 2017: per capita income less than or equal to R$ 85.00; years 2018 and 2019: per capita income less than or equal to R$ 89.00/month. Monetary values were updated based on the IPCA, considering the deflator calculated by the ratio of the December index of the current year in relation to the average of the indexes for each year, according to the update date. | Ministry of Citizenship/Secretariat for Evaluation and Information Management - SAGI | **X** | **X** |
| Percentage of self-declared brown or black people in the Single Registry (B_POPPRETAPARDACADS) | The indicator refers to the ratio between the self-declared black or brown population registered in the Cadastro Único and the total population in the Cadastro Único of the municipality, multiplied by 100. | Ministry of Citizenship/Secretariat for Evaluation and Information Management - SAGI. | **X** | **X** |
| Urbanization rate (D_POPPURB) | Ratio between the total number of people residing in the urban area of the municipality and its total resident population. For the years 2000 and 2010, population data are census data. For intercensal years, the population was estimated by interpolation. | IBGE/João Pinheiro Foundation. | **X** | **X** |
| Spending per capita on education activities (G_EDUCACAO) | Amount of budget expenditures presented in the Annual Rendering of Accounts ARA) carried out in the Elementary Education, Secondary Education, Professional Education, Higher Education, Kindergarten, Youth and Adult Education and Special Education subfunctions, divided by the total population of the municipality. | Court of Auditors of the State of Minas Gerais (TCE-MG). | **X** | **X** |
| Violent crime rate (P_CV) | Ratio between the number of occurrences, registered by the state police (military and civil), of violent crimes (Complete Homicide, Attempted Murder, Complete Robbery, Attempted Robbery, Extortion through Complete Kidnapping, Complete Kidnapping and Private Imprisonment, Kidnapping and Attempted Private Imprisonment, Completed Rape, Attempted Rape, Completed Vulnerable Rape, Attempted Vulnerable Rape, Completed Extortion and Attempted Extortion, as defined in Social Defense Event Records - REDS) and the population of the municipality; multiplied by 100,000. Notes: (1) As of 2019, the definition of "violent crimes" is composed of thirteen (13) criminal modalities, replacing the previous definition, composed of nine (09) modalities. (2) In 2020, data on "Violent Crimes" were updated, as of 2012, and correspond to the definition in force since 2019. | A) Public Security Observatory/Sejusp B) Total population: IBGE, Demographic Census of 2000 and 2010 (the population in the intercensus years was estimated by interpolation). | **X** | **X** |
| Rate of violent crimes against the person (P_CVPE) | Ratio between the number of occurrences, registered by the state police (military and civil), of crimes against the person (Complete Homicide, Attempted Murder, Completed Rape, Attempted Rape, Completed Vulnerable Rape and Attempted Vulnerable Rape, as defined in Records of Social Defense Events - REDS) and the population of the municipality; multiplied by 100,000. | A) Public Security Observatory/Sejusp B) Total population: IBGE, Demographic Census of 2000 and 2010 (the population in the intercensus years was estimated by interpolation). | **X** | **X** |
