# Supplementary material for: Association between contextual factors and coverage of the Acwy meningococcal vaccine, after three years of its overdue, in the vaccination calendar of adolescents in the state of Minas Gerais, Brazil: global space regressions
Source: BMC Infect Dis. 2023 Sep 19;23:615. doi: 10.1186/s12879-023-08549-6 (PMC10507822; doi:10.1186/s12879-023-08549-6)
Supplement: Supplementary file 2 — Additional file 2. [file 12879_2023_8549_MOESM2_ESM.docx]

| 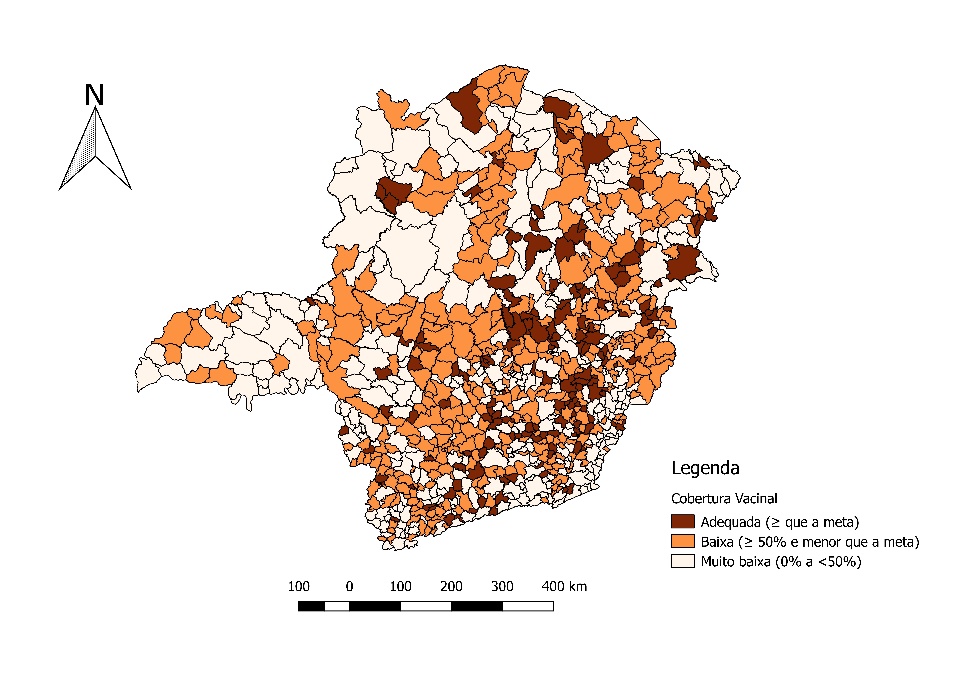   \| Legend  Vaccination coverage  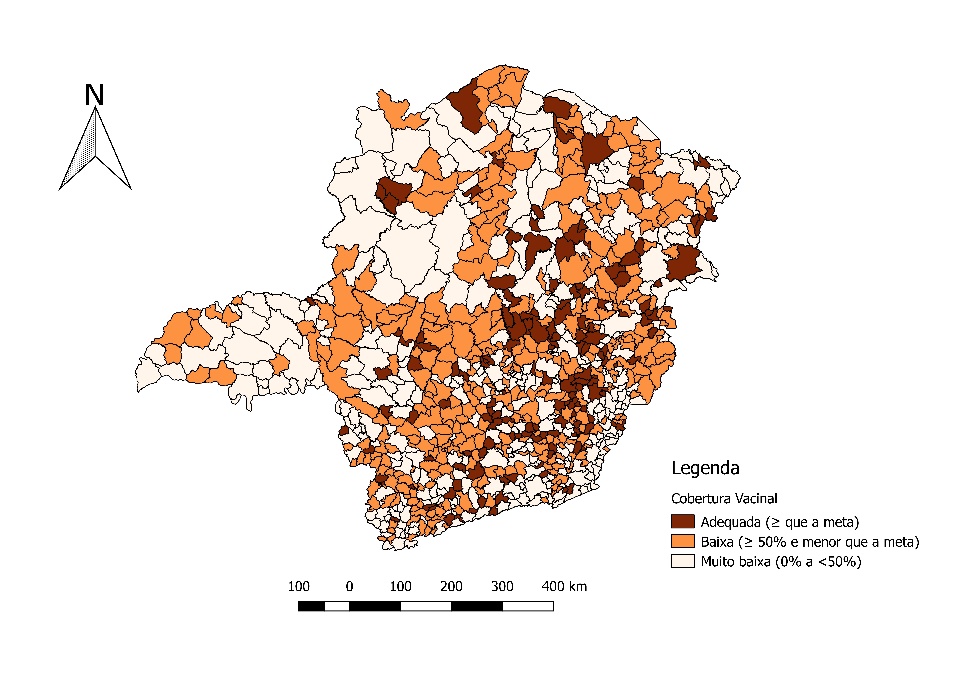  Adequate (≥ the target)  Low (≥ 50% and lower than the target)  Very low (0% a 5%) \| \| --- \|   A - 2020 |
| --- | --- |
| 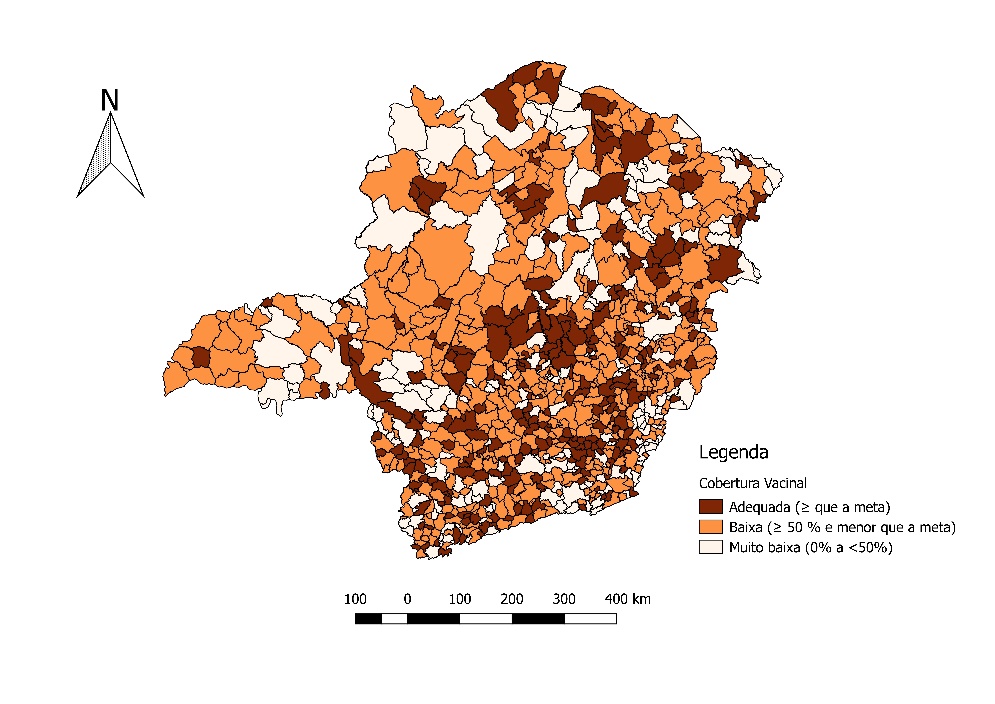   \| Legend  Vaccination coverage  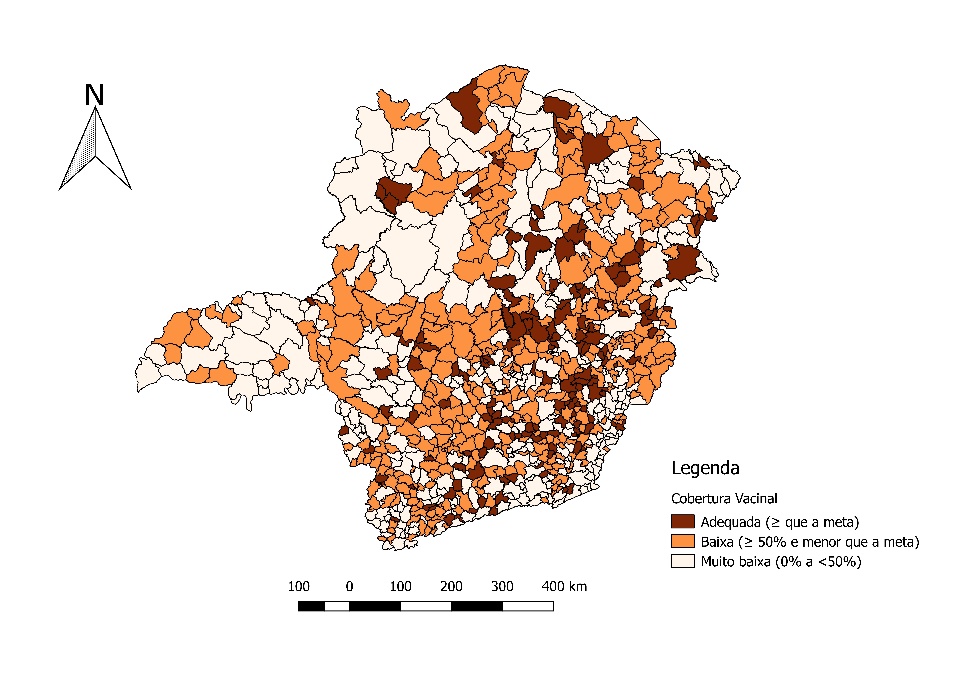  Adequate (≥ the target)  Low (≥ 50% and lower than the target)  Very low (0% a 5%) \| \| --- \|   B - 2021 |
| 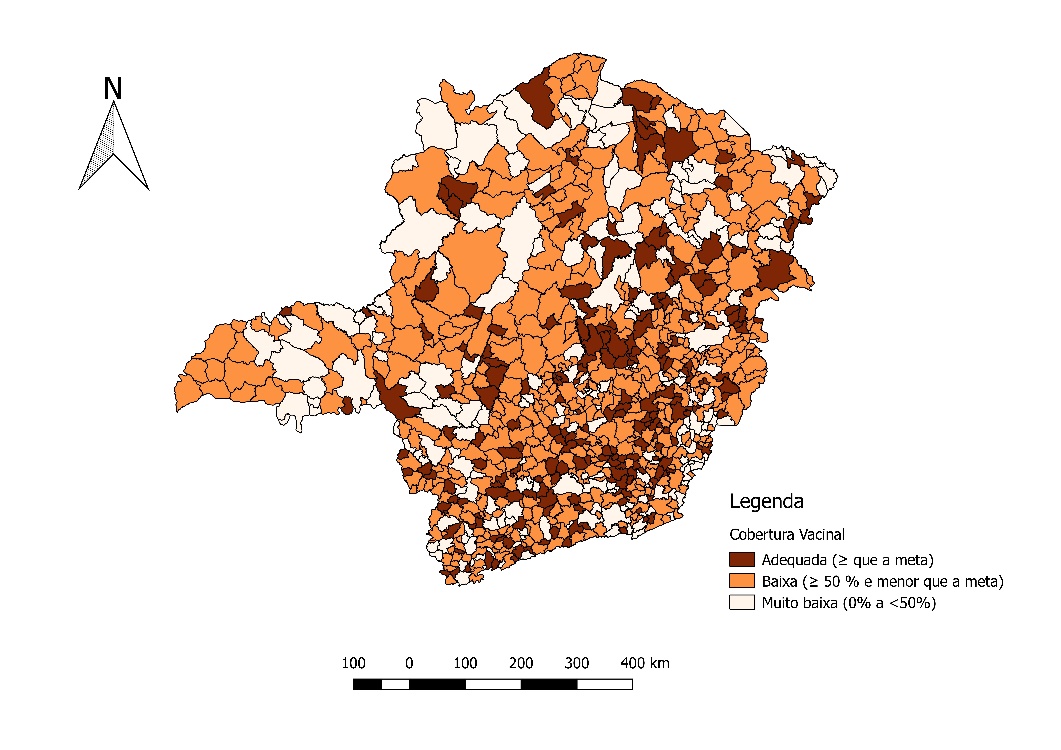  A   \| Legend  Vaccination coverage  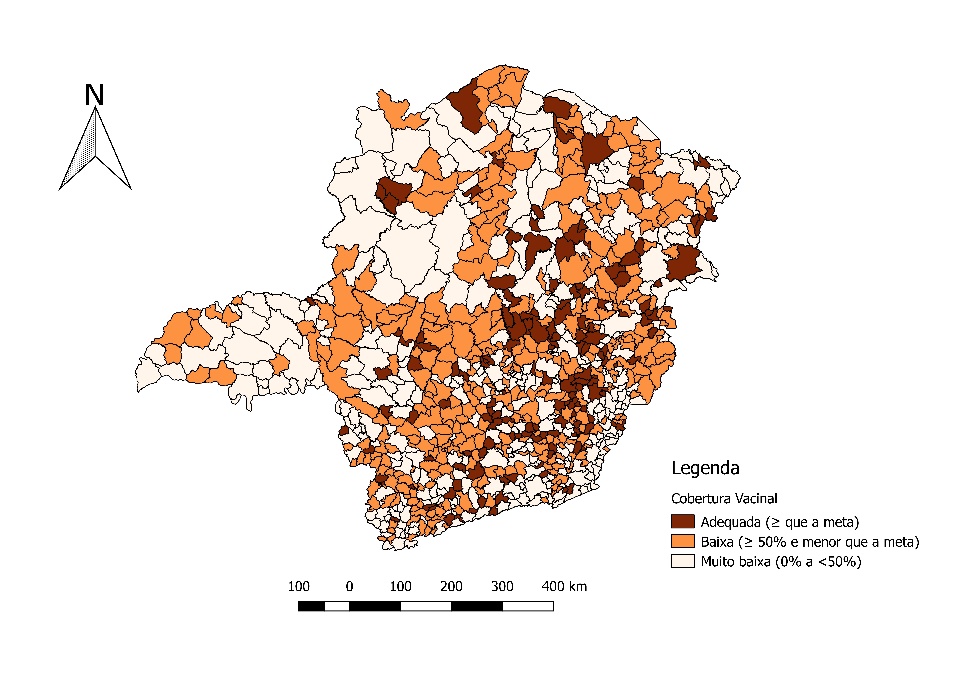  Adequate (≥ the target)  Low (≥ 50% and lower than the target)  Very low (0% a 5%) \| \| --- \|   C - 2022 |
| Suplementary 2 - Municipalities according to classification based on Meningococcal A, C, W and Y vaccine coverage, Minas Gerais, 2020 - 2022.  Note: 853 municipalities; goal ≥80%. |
