# Supplementary material for: Association between contextual factors and coverage of the Acwy meningococcal vaccine, after three years of its overdue, in the vaccination calendar of adolescents in the state of Minas Gerais, Brazil: global space regressions
Source: BMC Infect Dis. 2023 Sep 19;23:615. doi: 10.1186/s12879-023-08549-6 (PMC10507822; doi:10.1186/s12879-023-08549-6)
Supplement: Supplementary file 3 — Additional file 3. [file 12879_2023_8549_MOESM3_ESM.docx]

| 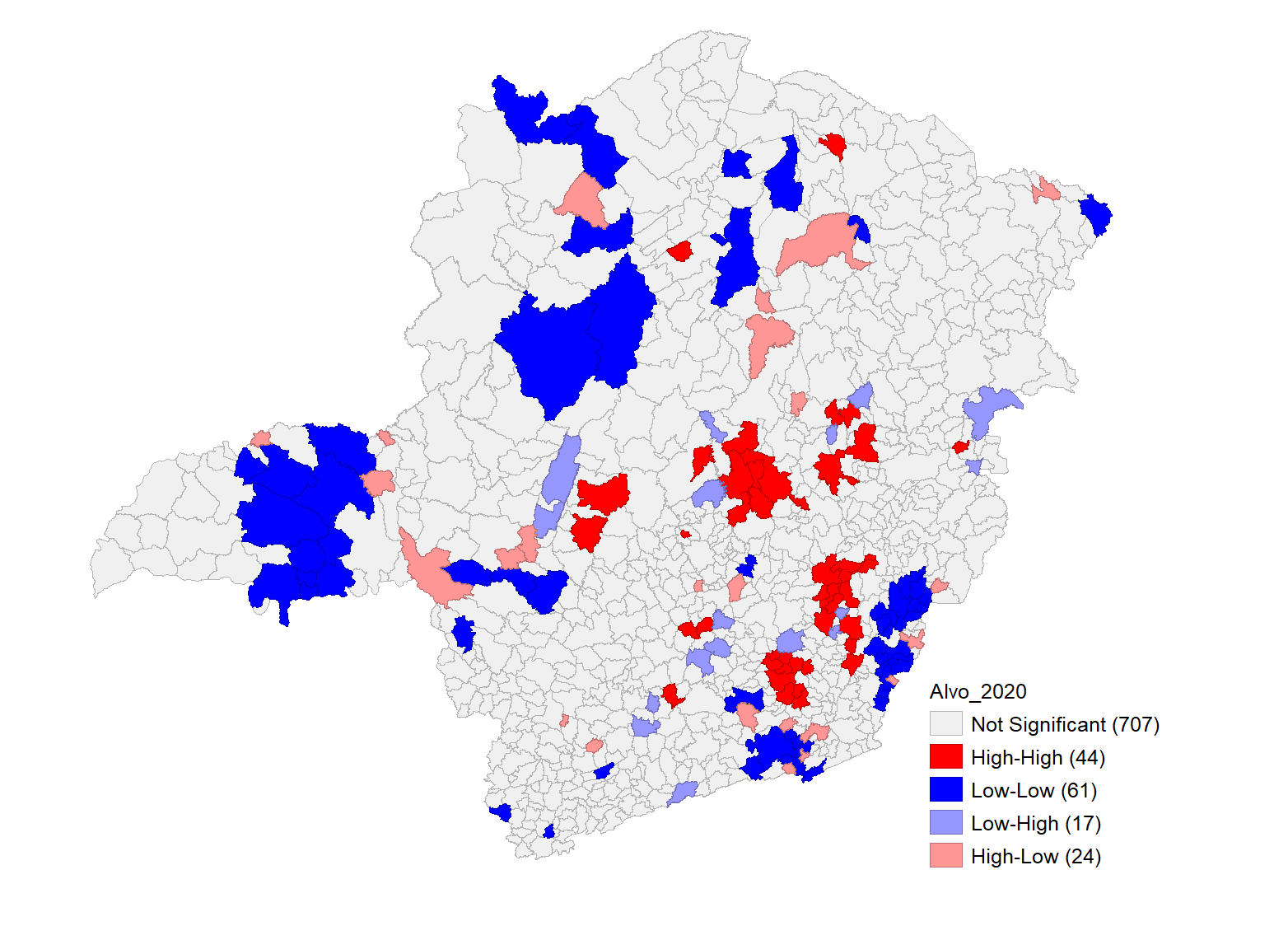  A - 2020 |
| --- |
| 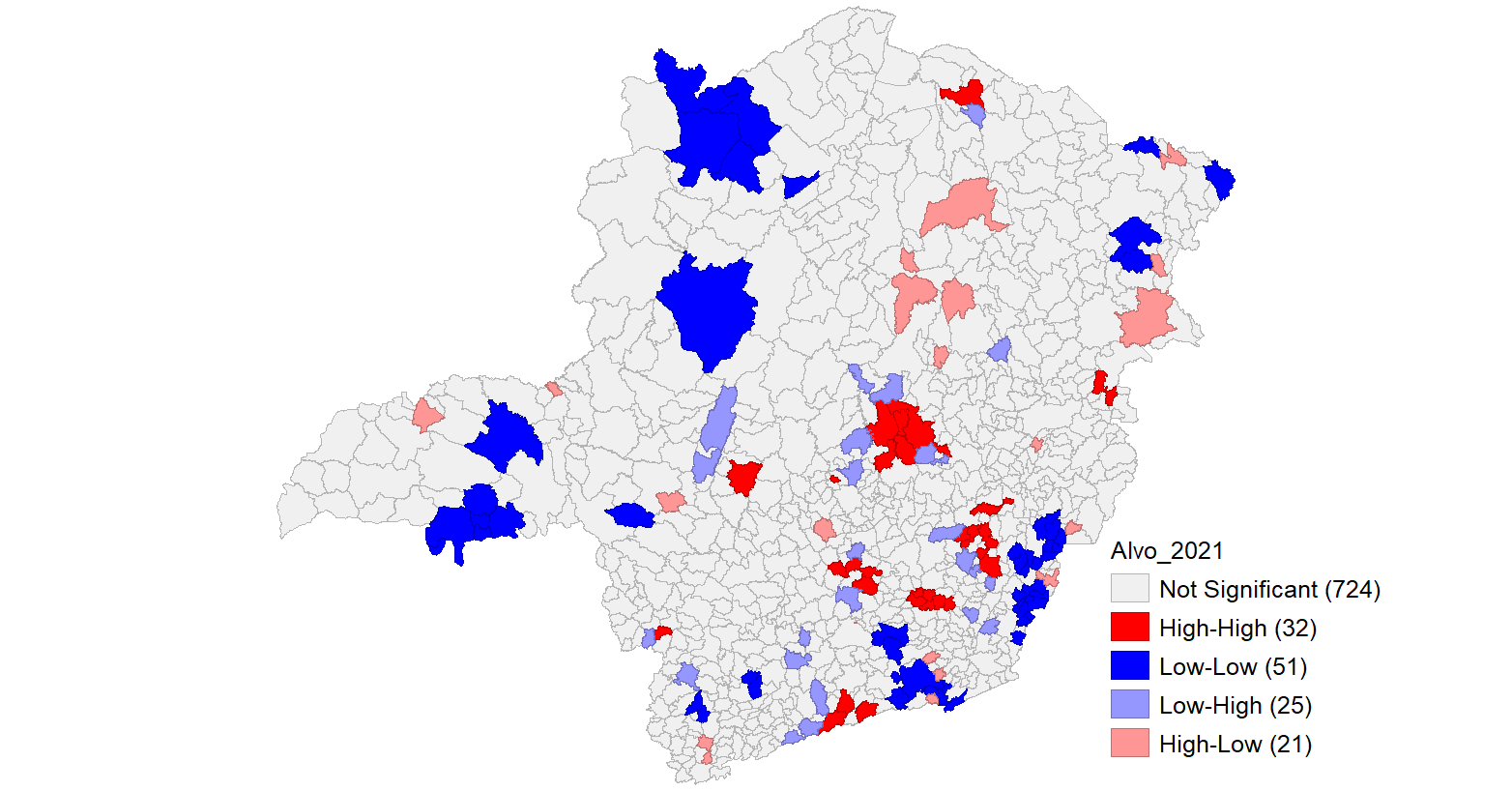  B - 2021 |
| 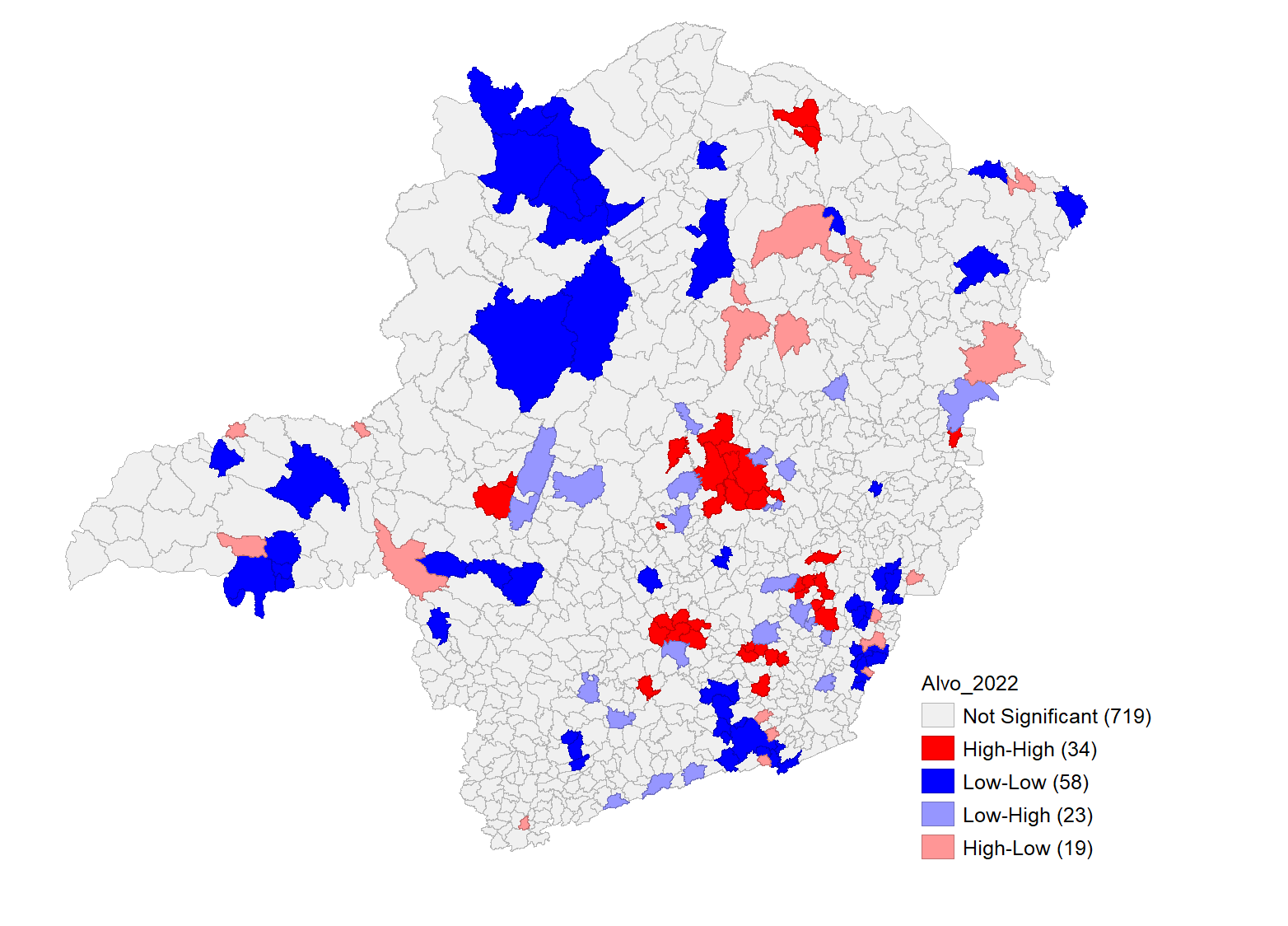  C - 2022 |
| Suplementary 3 - LISA Cluster Map showing the distribution of statistically significant spatial clusters of MenACWY vaccine coverage. Minas Gerais. 2020–2022.  Note: 853 municipalities. |
